# Effect of inulin and glycerol supplementation on physicochemical properties of probiotic frozen yogurt

**DOI:** 10.1080/16546628.2017.1290314

**Published:** 2017-03-03

**Authors:** Hafiz Shehzad Muzammil, Barbara Rasco, Shyam Sablani

**Affiliations:** ^a^ National Institute of Food Science Technology, University of Agriculture, Faisalabad, Pakistan; ^b^ School of Food Science, Washington State University, Pullman, WA, USA; ^c^ Biological Systems Engineering Department, Washington State University, Pullman, WA, USA

**Keywords:** Frozen yogurt, inulin, glycerol, glass transition temperature

## Abstract

The present study was designed to investigate the effects of inulin and glycerol supplementation on physicochemical properties of probiotic frozen yogurt. Frozen yogurt was prepared with different types of probiotic (*Lactobacillus acidophilus* and *Bifidobacterium lactis*) along with yogurt starter culture (*Streptococcus thermophilus* and *Lactobacillus bulgaricus*). The frozen yogurt mixture was supplemented with inulin (2%, 4%, and 6%) and glycerol (1%, 2%, 3%, and 4%). The results showed that inulin 4% and 6% supplementation increased the overrun by 3% and 5% and the glass transition temperature by 3.3% and 2.8%, and decreased the hardness by 7% and 11%, respectively. Inulin supplementation did not have a significant effect on ice crystal size (*p *> 0.05). Glycerol supplementation increased the stickiness from 2.4% to 18.7%, and decreased the hardness from 8.0% to 14.5% and the glass transition temperature from 2.4% to 34.5%, respectively. Glycerol supplementation did not have a significant effect on overrun or melting rate (*p *> 0.05).

## Introduction

Frozen yogurt is one of the most common types of functional food. People like and select functional foods not only because of their nutritional value but also for their therapeutic effects. Frozen yogurt is a unique dairy product with physical properties related to ice cream, and nutritional and sensory characteristics related to fermented milk products [1]. Other functional foods of dairy origin are bioyogurt, ice cream, frozen dairy desserts, fermented milks, various types of cheese, buttermilk, infant formula, concentrated milk, milk powder, and whey-based beverages [2]. Nowadays, novel varieties of frozen yogurt are produced, for example through supplementation with probiotics. Probiotics are live and beneficial microbes which, when administered in a sufficient quantity, help to improve the health of the consumers [3]. But when probiotics are incorporated into frozen products, their viability is a major concern. Mixing, fermentation, freezing, and overrun are the vital steps in the production of frozen yogurt, but these microbes are very sensitive to freeze injury, oxygen toxicity, and acidity [4–7], and these steps would result in the loss of viability in the frozen product. Various types of food ingredients and processing techniques have been investigated by researchers to improve probiotic viability in frozen products.

However, for food products to be acceptable to consumers, exceptional textural characteristics are most important. If a product is formulated to have good nutritional value but its textural quality is not as good as traditional ones, then its overall acceptability will be decreased [8].

Inulin is a polysaccharide belonging to the class fructans. It is not digested in the human gastrointestinal tract and it acts as dietary fiber. Beneficial microorganisms are able to use it as a growth substrate [9]. Chemically, inulin is a polymer with a fructose unit, β(2–1), linked to the terminal glucose unit. It can be obtained from the roots of various plants [10,11]. The degree of polymerization of inulin is 2–60 [12]. Inulin, being a complex carbohydrate, not only helps to increase the survivability of probiotics in frozen products, but also improves the textural properties of the products [13–16]. Furthermore, it can be used as a substitute for fat and sugar in food products without altering their structural and sensory properties [8,10,17–20].

Glycerol is a colorless, odorless, and sweet compound with cryoprotectant activity. It has three hydroxyl groups which form strong hydrogen bonds with water and improve its solubility. Frozen yogurt comprises about 60–70% water, depending on its formulation. During the freezing process, this water is converted into ice crystals. The shape and size of the ice crystals play a major role in the acceptability of the frozen product. The cryoprotectants also decrease the glass transition temperature and the solutions maintain a glassy state rather than freezing into a solid. Some cryoprotectants also bind with biological molecules as the water is removed, and maintain their structure and physiological functions.

Although the effects of inulin on structural and sensory properties have been investigated previously by many scientists, they used only low levels of inulin. Furthermore, the supplementation of glycerol as a cryoprotectant in frozen yogurt and its effect on the glass transition temperature have not been reported. Therefore, the present study was designed to prepare frozen yogurt with different levels of inulin and glycerol, and to evaluate their effects on various physiochemical properties, such as the glass transition temperature, overrun, melting rate, hardness, stickiness, air cells, and ice crystals.

## Material and methods

The yogurt starter culture YC-X 11® containing *Streptococcus thermophilus* and *Lactobacillus bulgaricus* and the probiotics *Lactobacillus acidophilus* LA-5 and *Bifidobacterium lactis* BB-12 (all in frozen direct vat set form) were obtained from Chr. Hansen laboratories (Chr. Hansen, Horsholm, Denmark).

The experiments were carried out at the Creamery (Milk Plant), School of Food Science and Human Nutrition, Washington State University (Pullman, WA, USA). A total 60 lb (27 kg) frozen yogurt mix was prepared by weighing and mixing 2.54 lb (1.15 kg) of skim milk powder, 6.68 lb (3 kg) of sugar (from a local grocery store), 8.36 lb (3.8 kg) of cream (40% milk fat; Dairy Gold, Seattle, WA, USA), and 0.3 lb (0.136 kg) of Ice Pro (Grindsted Ice Pro 20055 H, Stabilizer & Emulsifier System; Danisco, New Century, KS, USA) in 42.90 lb (19.48 kg) of milk, with continuous agitation at 45°C, followed by pasteurization at 80°C for 10 min and homogenization at 60°C in an APV Homogenizer (Model 400/200, MG-3 TPS; Gaulin, WI, USA) at 2000 psi in a single stage. After homogenization, one-quarter of this mixture was used to make the yogurt by adding yogurt starter culture and another quarter was used to propagate the probiotics by adding LA-5 and BB-12 at the rate of 0.02%, followed by incubation at 43°C for 4 h. Inulin (Orafti, LGI; Beneo-Orafti, Morris Plains, NJ, USA) was added as 2%, 4%, and 6%, respectively; then, each of the three parts was further divided into five parts, and food-grade glycerol (Sigma-Aldrich Co., St. Louis, MO, USA) was added at 0%, 1%, 2%, 3%, and 4%. Each of the mixtures was aged at 4°C for 24 h and then separately frozen in a batch ice-cream maker (Model 103; Taylor, Rockton, IL, USA) and packed in 4 oz (113 g) cups. The frozen yogurt was hardened at −35°C for 24 h and then stored at −20°C for further study.

## Physicochemical analysis of frozen yogurt

### Total solids and overrun

The total solids in the frozen yogurt mixture were determined by Official Method 990.19 of the Association of Analytical Chemists (AOAC) [21].

The amount of air incorporated was calculated by weighing the same volume of frozen yogurt before and after freezing in the same 500 ml container. The overrun was calculated by the formula [22]: Overrun% = [(*W*_1_ − *W*_2_)/*W*_2_] × 100, where *W*_1_ is the weight of frozen yogurt mix, and *W*_2_ is the weight of the same volume of frozen yogurt.

### Texture analysis

The texture of the frozen yogurt was measured after 1 week of frozen storage using a texture analyzer (TA.XT2; Stable Micro Systems, Godalming, Surrey, UK). A stainless steel cylindrical probe of 5 mm diameter attached to a 25 kg cell with a speed of 1 mm/s was allowed to penetrate to a depth of 10 mm at room temperature [1]. The hardness was measured as the force required for penetration, while the stickiness was measured as the force required for withdrawal of the probe.

### Microstructure

The microstructure of the frozen yogurt was analyzed by scanning electron microscopy (SEM) after freeze drying the samples. The frozen yogurt samples were cut into 6 cm cubes at −20°C and freeze dried in a freeze dryer (Vir-tis Freeze Mobile 24 with Uni-Top 600 l; Vir-Tis SP Industries Co., New York, NY, USA). The shelf temperature was −20°C, with a 20 Pa vacuum and a condenser temperature of −60°C. The samples were dried for 24 h. The freeze-dried samples were then analyzed by SEM (FEI 200 F Quanta; FEI Co., Hillsboro, OR, USA) at 20.00 kV and 250 × magnification. The analysis was carried out after 1 week of sample storage. The diameters of 250 air cells and ice crystals were measured with SEM software.

### Melting rate

The melting rate was calculated by taking a 60 g frozen yogurt sample and placing it on a wire mesh over a beaker at room temperature (23 ± 1°C). The melted frozen yogurt was collected in the beaker. After 90 min, the melted sample and residual sample were both weighed. After 1 week of frozen storage, the melting rate was calculated by dividing the mass of the melted sample over time (g/min).

### Glass transition temperature

The glass transition temperature (*T*_g_) was calculated with the help of a differential scanning calorimeter (DSC) (Q2000; TA Instruments, Newcastle, DE, USA) after the aging of the mixture. The instrument was calibrated with indium and sapphire by checking standard temperatures and enthalpies of fusion. Then, 10–20 mg of sample was sealed in an empty aluminum pan and placed in the sample chamber of the DSC along with an empty, sealed aluminum pan as reference. The sample in the pan was cooled from room temperature to −80°C at the rate of 5°C/min and equilibrated for 10 min at −80°C. Scanning of the sample was performed from −80°C to 50°C at the rate of 5°C/min. After scanning, the temperature was equilibrated at 25°C. The start, mid-, and end-points of glass transition were measured using TA Instruments Universal Analysis software.

The DSC draws a thermogram of heat flow versus temperature. The change in the heat-flow curve of the thermogram was represented as the glass transition temperature. The melting, endotherm peak area gave the enthalpy of melting (*H*_m_), which was calculated by drawing a linear baseline to the endotherm. The interaction of the baseline with the left side of the endotherm was taken as the end-point of freezing (*T*_m_). For annealing, the end-point of freezing (*T*_m_) was determined. In the second step, the same test was run with annealing using temperature *T*_m __−__ __1_ for 30 min. Here, 10–20 mg of sample was sealed in an empty aluminum pan and was cooled from room temperature to −80°C at the rate of 5°C/min and equilibrated for 10 min at −80°C. Annealing was performed at temperature *T*_m__ __−__ __1_ for 30 min by raising the temperature by 5°C/min. After annealing, scanning of sample was performed from −80°C to 50°C at the rate of 5°C/min and the temperature was equilibrated at 25°C. The start, mid-, and end-points of glass transition were measured using TA Instruments Universal Analysis software [23].

### Statistical analysis

All the data in the experiment were statistically analyzed by the SPSS statistical software program (version 19.0; IBM Corp., Armonk, NY, USA), using one-way analysis of variance. The comparison among treatment means was conducted using Duncan’s multiple range test at *p* < 0.05. The whole experiment was conducted twice and each test was run in triplicate.

## Results and discussion

### Total solids and overrun

The results in Table 1show that the total solids in the frozen yogurt samples increased significantly with the supplementation of inulin, while only 3% and 4% glycerol supplementation significantly increased the total solids compared with the control (*p* < 0.05).

The overrun values were low (Table 1) because the freezing process was carried out in a batch ice-cream maker. The increase in overrun with inulin 2% supplementation was not significant, whereas 4% and 6% supplementation showed significant increases in overrun (*p* < 0.05). This increase was recorded as 2.23%, 3.08%, and 4.98% with 2%, 4%, and 6% inulin supplementation, respectively. Although the addition of glycerol increased the overrun it was not significant, except for the samples containing 6% inulin with 3% and 4% glycerol (*p* < 0.05). Similar results were observed in previous studies [12,24,25]. Akalin and Erişir [12] observed an increase in overrun when they replaced dextrose corn syrup with inulin. Guven and Karaca [26] reported a 9% increase in overrun when they increased the sugar content by 4%. Karaca et al. [27] also observed an increase in overrun when inulin was used to replace fat. The increase in overrun may be due to the improvement in dry matter content and the interaction between inulin and some milk proteins forming a complex matrix in the frozen yogurt. This strong complex matrix, along with fat globules, may have entrapped more air bubbles, which were stabilized by fat globules, resulting in increased overrun percentage.

**Table 1.** Physicochemical properties of frozen yogurt with inulin (In) and glycerol (G).

		G 0%			G 1%			G 2%			G 3%			G 4%		
	Control	In 2%	In 4%	In 6%	In 2%	In 4%	In 6%	In 2%	In 4%	In 6%	In 2%	In 4%	In 6%	In 2%	In 4%	In 6%
Total solids (%)	30.04 ± 0.23^a^	32.02 ± 0.28^b^	34.03 ± 0.21^d^	34.03 ± 0.21^d^	32.16 ± 0.08^bc^	34.19 ± 0.10^de^	36.23 ± 0.22^fg^	32.23 ± 0.16^bc^	34.24 ± 0.19^de^	36.26 ± 0.27^fg^	32.34 ± 0.42^c^	34.37 ± 0.11^e^	36.37 ± 0.13^g^	32.45 ± 0.21^c^	34.37 ± 0.11^e^	36.46 ± 0.17^g^
Overrun (%)	27.02 ± 1.43^a^	29.25 ± 0.35^ab^	30.10 ± 0.81^b^	32.00 ± 1.27^b^	30.00 ± 0.99^b^	31.05 ± 1.20^b^	32.60 ± 0.85^bc^	31.00 ± 1.41^b^	31.00 ± 1.70^b^	34.55 ± 1.20^bc^	31.40 ± 1.42^b^	32.00 ± 1.27^b^	35.70 ± 1.56^c^	32.10 ± 1.84^b^	32.10 ± 1.84^b^	35.85 ± 1.34^c^
Hardness (N)	164.47 ± 5.82^abc^	184.71 ± 5.85^c^	152.74 ± 3.33^ab^	144.91 ± 2.96^ab^	170.80 ± 2.04^bc^	147.00 ± 2.82^ab^	138.00 ± 4.24^ab^	161.00 ± 1.41^abc^	140.00 ± 2.88^bc^	134.50 ± 3.54^bc^	155.00 ± 2.83^ab^	135.50 ± 3.54^bc^	128.68 ± 3.29^c^	147.50 ± 3.54^a^	127.00 ± 4.24^c^	123.00 ± 2.83^c^
Stickiness (N)	7.75 ± 0.17^a^	8.21 ± 0.13^b^	9.06 ± 0.26^e^	9.84 ± 0.57^h^	8.41 ± 0.05^c^	9.46 ± 0.18^g^	10.20 ± 0.42^i^	8.87 ± 0.22^d^	9.80 ± 0.77^h^	10.51 ± 0.57^j^	9.30 ± 0.34^f^	10.29 ± 0.57^i^	10.89 ± 0.85^l^	9.74 ± 0.15^h^	10.68 ± 0.09^k^	11.23 ± 0.64^m^
Melting rate (g/min)	0.39 ± 0.17^a^	0.37 ± 0.06^a^	0.35 ± 0.11^a^	0.32 ± 0.09^a^	0.40 ± 0.05^a^	0.37 ± 0.08^a^	0.36 ± 0.13^a^	0.44 ± 0.09^a^	0.40 ± 0.05^a^	0.40 ± 0.08^a^	0.49 ± 0.07^ab^	0.44 ± 0.13^a^	0.44 ± 0.05^a^	0.56 ± 0.04^b^	0.48 ± 0.08^ab^	0.47 ± 0.11^a^

Data are shown as mean ± SD.

^abc^ Means within the same column with different letters are significantly different (*p* < 0.05).

### Hardness and stickiness

The hardness of the frozen yogurt varied with different inulin and glycerol supplementation (Table 1). With 2% inulin supplementation, hardness increased by about 20.24 N, while in 4% and 6% supplemented samples it decreased by 11.73 N and 19.56 N compared to the control. Supplementation with 3% and 4% glycerol led to significant decreases in hardness (*p *< 0.05). The stickiness of the frozen yogurt (Table 1) increased significantly with inulin and glycerol supplementation (*p *< 0.05). The increase in stickiness was observed as 0.46 N, 1.31 N, and 2.09 N with 2%, 4%, and 6% inulin supplementation, respectively. These results were similar to those of El-Nagar et al. [18], who reported that the sugar and fat concentrations have opposite effects on the hardness of the ice cream. Karaca et al. [27] also observed a decrease in hardness with inulin supplementation. In our results, the decrease in hardness with the supplementation of inulin may be due to its water binding effect. Inulin forms a gel matrix within the product, reducing the availability of free water [11,16,18,24]. The glycerol acts as an emulsifier that decreases the hardness and increases the stickiness.

### Air cell size and ice crystal size

**Figure 1.**
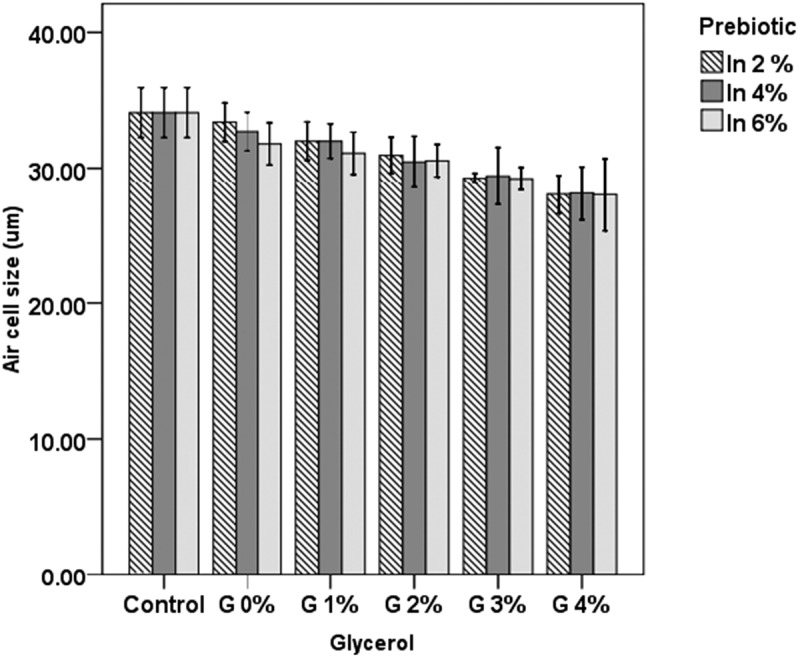
Air cell size in frozen yogurt with inulin (In) and glycerol (G).

The effects of inulin and glycerol supplementation are shown in Figure 1. Inulin supplementation significantly decreased the air cell size (*p *< 0.05), by 0.7 µm, 1.4 µm, and 2.29 µm with 2%, 4%, and 6% inulin, respectively. Glycerol supplementation also decreased the air cell size but this decrease was not significant except with 3% and 4% supplementation (*p *< 0.05). The small changes in air cell size may be due to the lower incorporation of air in the batch ice-cream maker and also due to the formation of a more viscous matrix with glycerol supplementation, which stabilized the air bubbles from collision.

**Figure 2.**
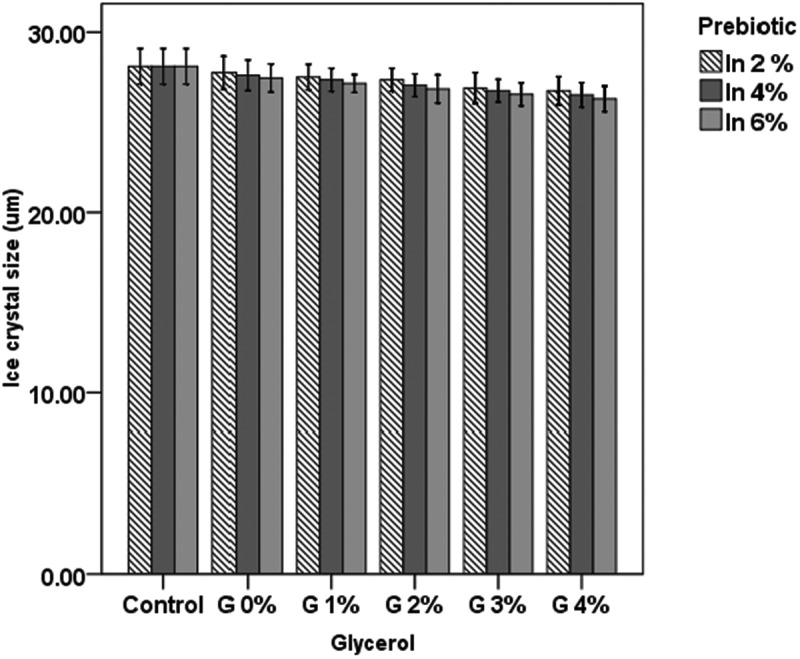
Ice crystal size in frozen yogurt with inulin (In) and glycerol (G).

Ice crystal size is shown in Figure 2. The results clearly show that the decrease in crystal size with inulin and glycerol supplementation was not significant (*p* > 0.05). The size range of the ice crystals was 26.31–28.15 µm in different samples. The ice crystal size was within an acceptable range in this study, whereas ice crystals in the range 40–55 µm would result in rough particles and decrease the sensory acceptability of the frozen product [22].

In the freezing process, the air is continuously beaten within the mixture and the temperature is reduced, leading to overrun and freezing of water. However, the resulting large air cells and ice crystals can be crushed into smaller ones by the impeller movement of the ice-cream maker, and stabilized by fat globules. The increased viscosity due to the addition of inulin and glycerol helped in the efficient breakdown of large air cells into smaller ones [28]. Furthermore, the inulin and glycerol supplementation may act as a cryoprotectant which binds much of the water, so that less free water is available for the development of large ice crystals. Therefore, this supplementation, along with quick freezing, prevented the formation of large ice crystals.

### Melting rate and glass transition temperature

The melting rate (Table 1) decreased significantly with inulin supplementation compared to the control, while glycerol supplementation showed a significant increase only in 2% inulin samples (*p *< 0.05). Similar results were reported by El-Nagar et al. [18] and Akin [29] (2005), who found a decrease in melting rate with the supplementation of inulin. This may be due to the high-molecular-weight inulin acting as a stabilizing agent and forming a sticky network. Thus, by immobilizing the water molecules, inulin may delay the melting of the frozen yogurt. The overrun percentage also influenced the melting rate. As air is a good insulator, the increased amount of overrun may also decrease the melting rate [25,30,31].

**Figure 3.**
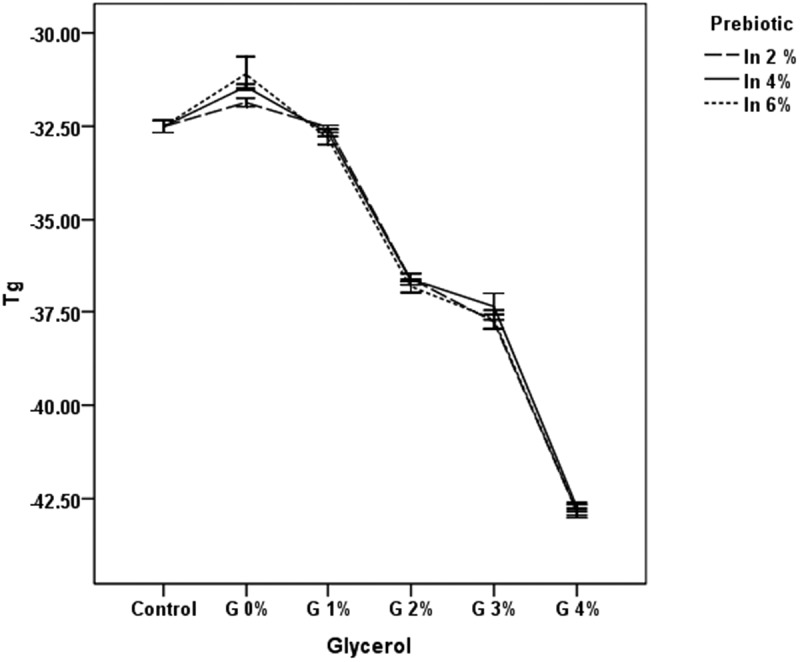
Glass transition temperature (*T*_g_) of frozen yogurt with inulin (In) and glycerol (G).

Inulin and glycerol act as cryoprotectants by affecting the glass transition temperature (Figure 3). Inulin supplementation increased the glass transition temperature while the addition of glycerol significantly decreased the glass transition temperature (*p *< 0.05). The glass transition temperature increased to 0.65ºC, 1.08ºC, and 1.42ºC with 2%, 4%, and 6% inulin supplementation, respectively. These results are similar to those of Soukoulis et al. [13], who reported increases in the glass transition temperature to 3.3°C and 4.2°C with 2% and 4% inulin supplementation. The addition of 1%, 2%, 3%, and 4% glycerol decreased the glass transition temperature to almost 1.0ºC, 5.0ºC, 6.0ºC, and 11.0ºC, respectively. The decrease in glass transition temperature may be due to glycerol’s unique properties. During rapid freezing, the viscosity of the glycerol as well as the aqueous media may increase. The glycerol molecules come closer to each other and form a three-dimensional arrangement through which water molecules can penetrate. The hydrogen bonds between the glycerol and water molecules are stronger than those bonding water–water molecules. All of these factors help to maintain the rubbery state of the yogurt ice cream, rather than its being glassy or solid at below freezing point. This decreased glass transition also affects the hardness and melting rate. The glycerol may prevent the development of large ice crystals that could have led to a decrease in hardness and increased the melting rate.

## Conclusion

The results of this study show that the supplementation of inulin and glycerol improved the physicochemical properties of frozen yogurt. The inulin increased the total solids, overrun, and stickiness, while its effects on the glass transition temperature and hardness were variable in prepared frozen yogurt. Inulin supplementation also decreased the melting rate, air cell size, and ice crystal size (*p *≤ 0.05). The glycerol improved the total solids and stickiness while lowering the hardness. Glycerol showed its greatest effect on the glass transition temperature, which was decreased to almost 34%. Supplementation with 3% and 4% glycerol showed greater effects on the improvement of physicochemical properties of the probiotic frozen yogurt (*p *≤ 0.05). Therefore, both inulin and glycerol can be used in functional and frozen dairy products to achieve good textural quality.
